# Performance of infectious diseases specialists, hospitalists, and other internal medicine physicians in antimicrobial case-based scenarios: Potential impact of antimicrobial stewardship programs at 16 Veterans’ Affairs medical centers

**DOI:** 10.1017/ice.2022.100

**Published:** 2023-03

**Authors:** Christopher J. Graber, Alissa R. Simon, Yue Zhang, Matthew Bidwell Goetz, Makoto M. Jones, Jorie M. Butler, Ann F. Chou, Peter A. Glassman

**Affiliations:** 1 Infectious Diseases Section, Veterans’ Affairs (VA) Greater Los Angeles Healthcare System, Los Angeles, California; 2 Center for Healthcare Innovation, Implementation and Policy, VA Greater Los Angeles Healthcare System, Los Angeles, California; 3 David Geffen School of Medicine, University of California–Los Angeles, Los Angeles, California; 4 IDEAS Center, VA Salt Lake City Healthcare System, Salt Lake City, Utah; 5 Department of Medicine, University of Utah, Salt Lake City, Utah; 6 Division of Epidemiology, University of Utah, Salt Lake City, Utah; 7 Department of Biomedical Informatics, University of Utah, Salt Lake City, Utah; 8 Department of Family and Preventive Medicine, College of Medicine, Oklahoma University Health Sciences Center, Oklahoma City, Oklahoma; 9 VA Pharmacy Benefits Management Services, Washington, DC

## Abstract

**Objective:**

As part of a project to implement antimicrobial dashboards at select facilities, we assessed physician attitudes and knowledge regarding antibiotic prescribing.

**Design:**

An online survey explored attitudes toward antimicrobial use and assessed respondents’ management of four clinical scenarios: cellulitis, community-acquired pneumonia, non–catheter-associated asymptomatic bacteriuria, and catheter-associated asymptomatic bacteriuria.

**Setting:**

This study was conducted across 16 Veterans’ Affairs (VA) medical centers in 2017.

**Participants:**

Physicians working in inpatient settings specializing in infectious diseases (ID), hospital medicine, and non-ID/hospitalist internal medicine.

**Methods:**

Scenario responses were scored by assigning +1 for answers most consistent with guidelines, 0 for less guideline-concordant but acceptable answers and −1 for guideline-discordant answers. Scores were normalized to 100% guideline concordant to 100% guideline discordant across all questions within a scenario, and mean scores were calculated across respondents by specialty. Differences in mean score per scenario were tested using analysis of variance (ANOVA).

**Results:**

Overall, 139 physicians completed the survey (19 ID physicians, 62 hospitalists, and 58 other internists). Attitudes were similar across the 3 groups. We detected a significant difference in cellulitis scenario scores (concordance: ID physicians, 76%; hospitalists, 58%; other internists, 52%; *P* = .0087). Scores were numerically but not significantly different across groups for community-acquired pneumonia (concordance: ID physicians, 75%; hospitalists, 60%; other internists, 56%; *P* = .0914), for non–catheter-associated asymptomatic bacteriuria (concordance: ID physicians, 65%; hospitalists, 55%; other internists, 40%; *P* = .322), and for catheter-associated asymptomatic bacteriuria (concordance: ID physicians, 27% concordant; hospitalists, 8% discordant; other internists 13% discordant; *P* = .12).

**Conclusions:**

Significant differences in performance regarding management of cellulitis and low overall performance regarding asymptomatic bacteriuria point to these conditions as being potentially high-yield targets for stewardship interventions.

The goal of antimicrobial stewardship is to promote proper antimicrobial therapy to improve not only the care of the patient at hand, including reducing or preventing complications such as *Clostridioides difficile*–associated colitis but also to preserve antimicrobial treatment options on individual and population levels in the future through the reduction of antimicrobial resistance.^1,2^ The Veterans’ Health Administration (VHA) has made a concerted effort to increase antimicrobial stewardship implementation over the past decade, starting with the charter of the VA Antimicrobial Stewardship Task Force in 2011 and a nationwide directive in 2014 that all VA facilities implement antimicrobial stewardship programs (ASPs),^3^ with much of the initial focus on inpatient care. Although reductions in inpatient antimicrobial use have been achieved,^3^ strategies are evolving regarding the involvement and education of inpatient providers who may have different training backgrounds, clinical experiences, and familiarity with antimicrobial stewardship principles. As part of a project implementing antimicrobial dashboards designed to provide feedback to antimicrobial stewards at select VA facilities nationwide, we assessed attitudes, knowledge, and prescribing practices regarding antimicrobial use and stewardship among different groups of physicians who typically provide inpatient care at VA facilities.

## Methods

The Cognitive Support Informatics for Antimicrobial Stewardship project enrolled 8 university-affiliated VA sites across the nation to participate in implementation of electronic antimicrobial dashboards that allow inter- and intrafacility comparisons of antimicrobial utilization across common inpatient conditions (eg, skin and soft-tissue infection, pneumonia, and urinary tract infection) over the duration of the typical hospital admission.^4^ To assess facility-level physician knowledge of appropriate antibiotic use, we administered an electronic survey via REDCap (www.project-redcap.org) to physicians who provide inpatient medical services at all 8 intervention sites along with 8 control sites, matched by complexity and geographic location, during October–December 2017. The full survey instrument is included in the Supplementary Materials (online). We contacted medical leadership at each participating facility to provide rosters of physicians who had provided inpatient acute general medicine services during the prior year. We invited those physicians to participate in the survey anonymously via e-mail. Over the course of 30 days, we sent 1 prenotification e-mail, 1 invitation with a survey link, and up to 8 reminder e-mails to initial nonrespondents. No incentives were provided for participation. The first portion of the survey collected information regarding physicians’ VA appointments, practice characteristics, attitudes toward antimicrobial use, and antibiotic prescribing practices. Questions about agreement with certain statements used Likert scales that were converted into numerical scores for analysis (1, strongly disagree; 2, disagree; 3, neutral; 4, agree; and 5, strongly agree). The second part of the survey explored how respondents would manage 4 clinical scenarios: cellulitis, community-acquired pneumonia (CAP), non–catheter-associated asymptomatic bacteriuria (NC-ASB), and catheter-associated asymptomatic bacteriuria (C-ASB). A final part of the survey addressed the availability and use of antibiotic prescribing resources.

For each scenario, responses were scored by assigning +1 for answers most concordant with Infectious Diseases Society of America guidelines at the time (ie, “correct”),^2,5–7^ 0 for less concordant but acceptable answers (or no answer given), and −1 for guideline-discordant answers (ie, “incorrect”). Guidelines were interpreted with emphasis on antimicrobial stewardship and practicality. One generalist (P.A.G.) and 2 infectious diseases physicians (C.J.G. and M.B.G.) collectively assigned a value to each answer to each subquestion a priori with free-text responses analyzed post hoc independent of knowledge of the type of practitioner giving the answer. For questions that allowed for multiple answers, 0 points were assigned when a less guideline-concordant answer was combined with a guideline-concordant answer, and −1 point was assigned when a guideline-discordant answer was combined with either a guideline-concordant or less guideline-concordant answer. Scores were then compiled across all questions within each scenario and were normalized from 100% concordant (all “correct”) to 100% discordant (all “incorrect”). Mean scores were calculated across respondents who self-identified as belonging to 1 of 3 categories: infectious diseases (ID) specialists, hospitalists, and other internists (general internal medicine and non-ID internal medicine subspecialists). For each question within a scenario, we tabulated percentages of responses based on the total number of survey participants in each physician category rather than the number in each category that responded to the individual question. Statistical significance of differences between groups were calculated using the Kruskal-Wallis rank-sum test, Pearson χ^2^ test, or the F test where appropriate. This study was approved by the Veterans’ Health Administration Central Institutional Review Board.

## Results

### Practice characteristics and antimicrobial attitudes, prescribing practices, and resource utilization

In total, 467 physicians who provided service on inpatient wards from all sites were contacted regarding participation in the survey. Among them, 159 answered at least 1 question (19 ID physicians, 71 hospitalists, and 69 other internists) and 140 respondents answered up to the first scenario (30.4% overall response rate): 19 ID physicians, 62 hospitalists, 58 other internists, and 1 respondent who did not identify a specialty. The respondent who did not identify a specialty was excluded, leaving 139 respondents to be analyzed. Of the 58 non-ID, nonhospitalist “other” internist respondents, 43 (74.1%) identified as generalists, 3 as rheumatologists, 3 as nephrologists, 2 as geriatricians, 2 as endocrinologists, 2 as pulmonologists, 2 as oncologists, and 1 as an endocrinologist and rheumatologist. No remarkable differences were identified between physician characteristics at intervention and control sites for any portion of the survey (data not shown). Practice characteristics and attitudes toward antimicrobial use are shown in Table 1. Significant differences were detected in proportion of time in clinical care (*P* = .023) and in inpatient care (*P* < .001), with hospitalists having the highest proportions. Attitudes toward antimicrobial use were largely similar across the 3 groups, though ID physicians more frequently felt that antibiotics were overused by clinicians at their facility (*P* = .002) and less likely felt that the harm of antibiotic overuse in livestock is exaggerated (*P* = .001). Of 139 physician respondents, 94 (67.6%) felt that antimicrobial stewardship programs were of at least moderate benefit to patient care at their institution, and 108 (77.7%) were satisfied or very satisfied with the assistance they have received from their facility regarding antibiotic prescribing over the prior year. Answers regarding antibiotic prescribing practices and resource utilization among provider groups are shown in Supplementary Table 1 (online). ID physicians were significantly more confident of their optimal use of antibiotics in the inpatient setting (*P* < .001) and were less likely to believe they may be overprescribing antibiotics in the inpatient setting (*P* = .019). ID physicians relied more on antibiograms (*P* = .017) than hospitalists and other internists in making antibiotic prescribing decisions. Numerically, they tended to rely less on electronic health record (EHR) templates (*P* = .144) and local infectious diseases online resources (*P* = .181) than the other 2 groups, but these differences were not statistically significant. Hospitalists and other internists frequently noted that they would find feedback on prescribing practices to be extremely or very helpful (82.3% hospitalists, 72.4% other internists, and 52.6% of ID physicians; *P* = .033), and hospitalists frequently noted that additional education or guidance on antibiotic prescribing would be extremely or very helpful (74.2% hospitalists, 46.6% other internists, and 26.3% of ID physicians; *P* < .001). Although non-ID respondents infrequently noted that their facility provided any new general guidance for antibiotic prescribing for skin and soft-tissue infection, pneumonia, and urinary tract infection across different time points of a typical hospital course, they frequently noted that the guidelines, when present, did influence their antibiotic prescribing practices (Supplementary Table 2 online). Among non-ID physicians, guidance regarding tailoring antibiotic courses after 3 days affected antibiotic prescribing practices for pneumonia (97.1%) significantly more frequently than guidance regarding skin and soft-tissue infection (80%) or urinary tract infection (90%) (*P* = .0079). No significant differences across these conditions were detected for guidance regarding initial choice and completion of an antibiotic course.

**Table 1.** Practice Characteristics and Attitudes Toward Antimicrobial Use Among Survey Respondents (by Specialty)

Characteristic	ID (n = 19)	Hospitalists (n = 62)	Other Internists (n = 58)
**Demographics**			
**Age**			
≤25 y, no. (%)	(0)	0 (0)	0 (0)
26–35 y, no. (%)	5 (26.3)	17 (27.4)	11 (19)
36–45 y, no. (%)	5 (26.3)	25 (40.3)	16 (27.6)
46–55 y, no. (%)	3 (15.8)	10 (16.1)	12 (20.7)
56–65 y, no. (%)	2 (10.5)	4 (6.5)	9 (15.5)
>65 y, no. (%)	3 (15.8)	0 (0)	1 (1.7)
No answer, no. (%)	1 (5.3)	6 (9.7)	9 (15.5)
**Sex**			
Male, no. (%)	14 (73.7)	30 (48.4)	27 (46.6)
Female, no. (%)	3 (15.8)	27 (43.5)	22 (37.9)
**Practice characteristics**			
Years since completing clinical training, median (range)	6 (1–46)	7 (0–34)	13 (0–38)
Years practicing within VA, median (range)	6 (1.5–46)	5 (0.5–23)	7 (0.5–38)
Full-time practice at VA, no. (%)	14 (73.7)	45 (72.6)	46 (79.3)
% Time spent in clinical care, median (range)*	50 (15–100)	65 (0–100)	50 (0–100)
% Clinical time spent on inpatient care, median (range)**	75 (33–100)	100 (0–100)	40 (2–100)
**Attitudes toward antimicrobial use, Likert** ^**a**^ **mean (SD)**			
Antibiotics are overused nationally	4.74 (0.45)	4.63 (0.48)	4.44 (0.66)
Antibiotics are overused by clinicians at my facility*	4.32 (0.67)	3.63 (0.73)	3.72 (0.77)
Better use of antibiotics will reduce problems with antibiotic-resistant organisms	4.63 (0.50)	4.53 (0.59)	4.60 (0.53)
Strong knowledge of antibiotics is important in my medical career	4.84 (0.37)	4.74 (0.44)	4.55 (0.57)
Prescribing broad-spectrum antibiotics when equally effective narrower-spectrum antibiotics are available increases antibiotic resistance	4.53 (0.61)	4.56 (0.64)	4.59 (0.56)
Hand washing/cleaning practices are not utilized to the recommended extent at my facility	3.37 (1.30)	2.73 (1.16)	2.59 (1.08)
Inappropriate use of antibiotics can harm patients	4.84 (0.37)	4.72 (0.49)	4.62 (0.53)
The harm of antibiotic overuse in livestock is exaggerated*	1.37 (0.50)	1.87 (0.82)	2.16 (0.81)
The harm of antibiotic overuse in humans is exaggerated	1.58 (0.77)	1.58 (0.76)	1.81 (0.85)
**At your facility, how much of an obstacle or benefit are antimicrobial stewardship programs to good patient care?**			
Considerable benefit, no. (%)	6 (31.6)	24 (38.7)	22 (37.9)
Moderate benefit, no. (%)	9 (47.4)	20 (32.3)	13 (22.4)
Others, no. (%)	4 (21)	18 (29)	23 (39.7)
**What is your overall satisfaction with the assistance you have received from your facility regarding antibiotic prescribing for your inpatients over the past 12 months?**			
Very satisfied, no. (%)	8 (42.1)	26 (41.9)	23 (39.7)
Satisfied, no. (%)	8 (42.1)	22 (35.5)	21 (36.2)
Others, no. (%)	3 (15.8)	14 (22.6)	14 (24.1)

Note. VA, Department of Veterans’ Affairs.

*P < .05; **P < .001.

a Likert scale: 1, strongly disagree; 2, disagree; 3, neutral; 4, agree; 5, strongly agree.

### Clinical scenario performance

Clinical scenario scores are summarized in Table 2, with full descriptions of each scenario and all responses are listed in Supplementary Table 3 (online). Scenario 1 describes a case of simple spreading of cellulitis in the lower extremity with blood cultures on admission that turned positive for group A *Streptococcus*. We detected a significant difference in scores for this scenario (ID physicians, 76% concordant; hospitalists, 58% concordant; other internists, 52% concordant; *P* = .0087), driven mostly by differences in appropriately classifying the clinical condition as cellulitis alone (*P* = .019). Scenario 2 describes a case of community-acquired pneumonia in which high-quality respiratory cultures grow *Streptococcus pneumoniae*. Scores were numerically but not significantly different across specialties for this scenario (ID physicians, 75% concordant; hospitalists, 60% concordant; other internists, 56% concordant; *P* = .0914), though ID physicians were significantly more likely to select appropriate oral antimicrobial therapy on day 3 (*P* = .006). Scenarios 3 and 4 presented cases of asymptomatic bacteriuria (in a noncatheterized patient in scenario 3 and a catheterized patient in scenario 4), and 2 questions were given for each scenario: (1) What is the clinical presentation? The guideline-concordant answer was asymptomatic bacteriuria. And (2) what is the antibiotic treatment? The guideline-concordant answer was none. All specialties (including infectious diseases) did poorly on both scenarios, with ID physicians answering 65% concordant, hospitalists 55% concordant, and other internists 40% concordant (*P* = .322) on scenario 3. For scenario 4, ID physicians answered 27% concordantly, but hospitalists and other internists actually had mean negative scores of 8% (consistent with guideline discordance) and other internists had mean negative scores of 13% (*P* = .12). Other internists were more likely to incorrectly select an antibiotic in scenario 3 (*P* = .034).

**Table 2.** Summary of Clinical Scenario Scores by Practice Type

Variable	Average Points		
	ID (n = 19)	Hospitalists (n = 62)	Other Internists (n = 58)
**Scenario 1: Simple cellulitis**			
Syndrome classification*	+1.0	+0.65	+0.69
Initial antibiotic choice	+0.53	+0.21	+0.34
De-escalation antibiotic choice	+0.84	+0.65	+0.45
Discharge antibiotic choice	+0.84	+0.71	+0.52
Treatment duration	+0.58	+0.68	+0.60
Scenario 1 total points (*P =* .0087 by F test)*	+3.8 (76% concordant)	+2.9 (58% concordant)	+2.6 (52% concordant)
**Scenario 2: Community-acquired pneumonia**			
Syndrome classification	+0.74	+0.61	+0.69
Initial antibiotic choice	+0.58	+0.56	+0.52
De-escalation antibiotic choice*	+0.74	+0.34	+0.33
Treatment duration	+0.90	+0.89	+0.69
Scenario 2 total points (*P =* .091 by F test)	3.0 (75% concordant)	2.4 (60% concordant)	2.23 (56% concordant)
**Scenario 3: Asymptomatic bacteriuria (non–catheter associated)**			
Syndrome classification	+0.63	+0.56	+0.41
Antibiotic choice*	+0.63	+0.52	+0.34
Scenario 3 total points (*P =* .322 by F test)	1.3 (65% concordant)	1.1 (55% concordant)	0.8 (40% concordant)
**Scenario 4: Asymptomatic bacteriuria (catheter associated)**			
Syndrome classification	0	−0.18	−0.21
Antibiotic choice	+0.53	+0.016	−0.052
Scenario 4 total points (*P* = .12 by F test)*	0.53 (27% concordant)	−0.16 (8% discordant)	−0.26 (13% discordant)

Note. ID, infectious disease.

*P < .05.

Physicians were asked after each scenario what resources they would most likely use in management of the case at hand. General medical resources (eg, UpToDate or a medical textbook) were most frequently selected, though prespecified guidance from the facility and information and input from an inpatient ward pharmacist were also commonly selected (Supplementary Table 4 online). After each scenario, physicians were asked about their confidence in making antibiotic prescribing decisions for the patient in the scenario without the use of those resources. ID physicians were significantly more confident in all scenarios, particularly for scenario 1 (the cellulitis scenario; 84.2% “very confident” vs 27.4% for hospitalists and 39.7% for other internists; *P* < .001) (Supplementary Table 5 online), but confidence did not correlate with performance (data not shown).

Finally, we examined whether non–ID physician awareness of new guidance within the prior 12 months from their facilities’ ID division or antimicrobial stewardship team on the initial choice, tailoring, and completion of an antibiotic course was associated with their performance on the clinical scenarios. Although no significant associations were detected for hospitalists, other internists’ overall awareness of this guidance was associated with higher performance across all scenarios (*P* = .011), driven mostly by awareness of guidance regarding management of pneumonia (*P* = .001, data not shown).

## Discussion

We detected significant differences in survey responses between ID physicians, hospitalists, and generalists on how to manage infectious conditions that are commonly seen in the practice of inpatient internal medicine and are frequently targets for antimicrobial stewardship interventions. Most notably, the low overall scores in management of asymptomatic bacteriuria (both non–catheter-associated and catheter-associated) point to the difficulties inherent in recognizing and/or avoiding antimicrobial treatment for this situation and the need for education and interventions in this domain that target all physicians who practice inpatient internal medicine, even ID physicians. Implementation of algorithm-based peer feedback has been shown to be successful in this regard.^8^ A knowledge gap between ID physicians and other specialties on the management of cellulitis also points to opportunities for developing stewardship interventions targeted at non-ID physicians and focusing on the management of skin and soft-tissue disease. All specialties scored highest on the community-acquired pneumonia scenario, but opportunities exist for improvement in this domain as well. As with cellulitis, de-escalation of antimicrobial therapy when culture data return and the patient is improved clinically was a particular weak point, indicating an opportunity for targeted interventions.

Specialties likely differ in terms of how they can best be targeted by stewardship interventions. A recent study of inpatient services at an academic medical center demonstrated that generalist-led services prescribed more broad-spectrum therapy than hospitalist-led services.^9^ In our survey, hospitalists and other internists tended to rely less on antibiograms than ID physicians in their clinical practice. Although hospitalists and other internists tended to rely more on EHR templates and local infectious diseases online resources, overall reliance on these modalities was low. This finding illustrates an antimicrobial stewardship principle that occurs frequently in the literature: educational or informational resources make an impact when accompanied by patient-level antimicrobial stewardship team intervention.^10–14^ More involvement of antimicrobial stewardship teams in provider-facing activities, such as audit and feedback and in-person presence on rounds (“handshake stewardship”), may be particularly effective.^2,15–19^ Physicians in our survey indicated that online general medical resources (eg, UpToDate, Wolters Kluwer) are the most frequently referenced when making antibiotic prescribing decisions. Antimicrobial stewards should routinely ensure that these resources reinforce antimicrobial prescribing principles at their facilities. Hospitalists and other internists particularly noted a desire for more feedback on prescribing practices, signifying awareness of their knowledge gaps and interest in improving upon them. Other internists seemed particularly influenced by guidance on antimicrobial prescription for pneumonia, particularly when tailoring therapy around hospital day 3.

Our study had several limitations. A low number of ID physician respondents significantly limited our ability to make inferences about the ID community at large. The overall response rate was also relatively low. The survey was lengthy; not all respondents answered all questions, and there may be a bias toward those who had more available time, altruism, or interest in the subject. Although clinical scenarios can be effective in demonstrating physician proficiency independent of patient case mix and other factors that may influence patient care-related metrics,^20^ our scenarios may have been worded in a way that was less clear or not fully representative of real-life circumstances. For example, we noted in the community-acquired pneumonia case that the patient presented “from home” but did not give details that further suggested community versus healthcare-associated acquisition. Factors such as this may have influenced respondents to invoke underlying biases and experiences that may not truly reflect antibiotic prescribing expertise. Finally, the small number of questions pertaining to management of asymptomatic bacteriuria increased the variance in our estimate of provider understanding of its management. However, the overall detailed information we received on antimicrobial prescribing practices should serve as a useful roadmap for stewards who are trying to balance the attitude, knowledge, and practice differences of the practitioners at their facility in planning antimicrobial stewardship interventions.

## Supplementary material

For supplementary material accompanying this paper visit https://doi.org/10.1017/ice.2022.100. click here to view supplementary material
