# Supplementary material for: Performance of infectious diseases specialists, hospitalists, and other internal medicine physicians in antimicrobial case-based scenarios: Potential impact of antimicrobial stewardship programs at 16 Veterans’ Affairs medical centers
Source: Infect Control Hosp Epidemiol. 2022 May 4;44(3):400–5. doi: 10.1017/ice.2022.100 (PMC10015262; doi:10.1017/ice.2022.100)
Supplement: Supplementary file 1 [file S0899823X22001003sup.zip › S0899823X22001003sup001.docx]

**Clinician Antibiotic Prescribing (CAP) Survey**

**About this survey:**

This survey is being conducted as part of a research project entitled *Cognitive Support Informatics for Antimicrobial Stewardship,* Peter Glassman, Principal investigator (PI). The survey asks about your antibiotic prescribing experiences and perspectives, to better understand the impact of antimicrobial stewardship programs (ASPs) across VA.

**About your participation:**

This survey is strictly voluntary; your participation decision will have no adverse effect on you professionally or personally. All responses will remain completely confidential. Answering survey questions will constitute agreement to participate. After the survey period has ended, each respondent will receive aggregated survey results. For more detailed information about the survey and your participation, please refer to the Question and Answer addendum which you can download by clicking on the Microsoft Word file at the bottom of this page.

**Completing and returning your survey:**

Complete the survey by clicking on the appropriate answers, and using the spaces provided to write in responses and comments. When you have completed the survey, make sure you click on the "Submit" button on the last page to record your responses.

If you have questions or wish to discuss participation, please contact the project PI, Dr. Peter Glassman at peter.glassman@va.gov. If you have technical difficulties with the survey, contact the survey administrator, Alissa Simon, at [alissa.simon@va.gov](mailto:alissa.simon@va.gov) or 818-891-7711 x36063.

[WORD FILE HERE]

**Professional Background and Practice Characteristics**

1. During the past six months, have you spent at least two weeks as an attending physician on general medicine inpatient service at a VAMC?

Check one

- Yes **⇨** Please continue
- No/Not a staff physician **⇨** Thank you for your response. We do not need you to complete the remainder of this survey.

1. What is your medical specialty, or area(s) of practice?

Check all that apply

- General Internal Medicine
- Family Medicine
- Hospitalist
- Infectious disease specialist
- Geriatrician
- Other Internal Medical Subspecialist (Specify) _______________________________________
- Other/None of the above **⇨** Ineligible text

1. What year did you complete your clinical training (e.g., highest level of residency/fellowship)?

Enter 4 digit year

1. How many years total have you been practicing at the VA? Include all years if at multiple VAs or multiple time periods. Exclude training.

Enter total #/years

1. How many years have you been practicing at VA?
   Include all years if at multiple VAs or multiple time periods. Exclude training.

Enter #/years-use decimals for partial years

1. How many eighths is your current VA appointment?

Check one

- 1/8  2/8  3/8  4/8  5/8  6/8  7/8  8/8

**Questions in this survey ask about your current VA facility, that is [FACILITY].**

1. If [FACILITY] is not your primary VA facility, please specify here and answer about that facility.

Enter #/years-use decimals for partial years

1. How many years have you been practicing at your current facility?.
2. Within the past 12 months, did you practice medicine in an inpatient setting outside of VA?

Check one

- Yes **⇨**
- No

1. Do you currently have a university affiliation?

Check one

- Yes
- No

1. During the past year, on average, how much of your time was spent in the following activities?
   Your best estimate is fine

|  | % |
| --- | --- |
| Clinical care |  |
| Research activities |  |
| Education/teaching activities |  |
| Administrative activities |  |
| Other (specify) ___________________ |  |
| **Total:** | 100% |

1. During the past year, what proportion of your clinical time was spent on inpatient vs. outpatient care?

|  | % |
| --- | --- |
| Inpatient |  |
| Outpatient |  |
| **Total:** | 100% |

1. During the past year, how many weeks did you attend on the following VA services?

|  | Enter #/weeks |
| --- | --- |
| General medicine inpatient service |  |
| ICU or CCU (or a respective step down unit) |  |
| Transitional care unit or similar |  |
| Geriatric unit |  |
| Community Living Center (CLC) |  |
| Inpatient consult service (specify: ______) |  |
| Other inpatient service (specify: ______) |  |

1. When you attended on a general medicine ward team on the VAMC inpatient service, were any of the following typically part of your team?

Check all that apply

- Pharmacist
- Physician Trainee (e.g., resident, fellow)
- Medical or other student
- Nurse Practitioner or Physician Assistant
- None of the above (exclusive answer)

1. Of the patients you cared for on your last general medicine inpatient rotation, approximately what proportion were admitted for a primary infectious disease diagnosis?

Check one

- 0-25%
- 26-50%
- 51-75%
- 76-100%

1. Of the patients you managed during your last general medicine inpatient rotation, please estimate what proportion were admitted from the following?

|  | % |
| --- | --- |
| Emergency Department |  |
| VA Primary Care |  |
| VA Specialty Care (inpatient transfer or from an outpatient clinic) |  |
| Transfer from another hospital |  |
| ICU transfer |  |
| Other (specify) ___________________ |  |
| **Total:** | 100% |

We would like to know about ID consults and interactions with Antimicrobial Stewardship Program (ASP) personnel regarding your inpatients admitted with an infectious disease diagnosis..

1. For what proportion of your inpatients with an infectious disease diagnosis in the last 12 months did you request or receive input, either formal or informal, from an ID consult service?

Check one

- 76-100% of patients
- 51-75% of patients
- 26-50% of patients
- 1-25% of patients
- 0%

**Attitudes Relating to Antibiotic Use**

1. Please indicate your agreement or disagreement with the following statements about antibiotic use.

|  | Strongly disagree | Disagree | Neither agree nor disagree | Agree | Strongly agree |
| --- | --- | --- | --- | --- | --- |
|  | Check one on each line | | | | |
| Antibiotics are overused nationally | □ | □ | □ | □ | □ |
| Antibiotics are overused by clinicians at my facility | □ | □ | □ | □ | □ |
| Better use of antibiotics will reduce problems with antibiotic-resistant organisms | □ | □ | □ | □ | □ |
| Strong knowledge of antibiotics is important in my medical career | □ | □ | □ | □ | □ |
| Prescribing broad spectrum antibiotics when equally effective narrower-spectrum antibiotics are available increases antibiotic resistance | □ | □ | □ | □ | □ |
| Hand washing/cleaning practices are not utilized to the recommended extent at my facility | □ | □ | □ | □ | □ |
| Inappropriate use of antibiotics can harm patients | □ | □ | □ | □ | □ |
| The harm of antibiotic overuse in livestock is exaggerated | □ | □ | □ | □ | □ |
| The harm of antibiotic overuse in humans is exaggerated | □ | □ | □ | □ | □ |

1. How much of a problem, if any, do you believe antibiotic resistance is in the following settings?

|  | Formidable problem | Considerable problem | Moderate problem | Minor problem | Not a problem | |
| --- | --- | --- | --- | --- | --- | --- |
|  | Check one on each line | | | | | |
| Nationally | | □ | □ | □ | □ | □ |
| In your community | | □ | □ | □ | □ | □ |
| In your VA medical center | | □ | □ | □ | □ | □ |
| In your VA practice or on your VA service | | □ | □ | □ | □ | □ |

**Antibiotic Prescribing Practices**

1. How confident are you that you use antibiotics optimally in the inpatient setting?

Check one

- Very confident
- Somewhat confident
- Not very confident
- Not at all confident

1. In general, for hospitalized patients who are admitted with a diagnosis of either uncomplicated healthcare-acquired pneumonia, non-diabetic cellulitis, or uncomplicated UTI (cystitis), how important do you feel it is to reassess the antibiotic regimen on or around day 3 to tailor therapy?

Check one

- Very important
- Somewhat important
- Not very important
- Not at all important

1. For patients that were admitted to your care for an infectious etiology during the past 12 months, how often did you do the following?

|  | Always/ most often  (81-100%) | Usually  (61-80%) | About half the time  (41-60%) | Sometimes  (21-40%) | Never/ not often  (0-20%) |
| --- | --- | --- | --- | --- | --- |
|  | Check one on each line | | | | |
| Change the antibiotics that were initially started by the ER (usually on day 1 or 2 of hospitalization). That is, adjust the antibiotic regimen soon after admission. | □ | □ | □ | □ | □ |
| Tailor the antibiotic regimen on or around day 3 if appropriate. That is, narrow the antibiotic regimen after the patient has been hospitalized for several days. | □ | □ | □ | □ | □ |
| Observe the patient on oral antibiotics for at least one day before discharge. | □ | □ | □ | □ | □ |
| Continue with an IV antibiotic until within 1 day of anticipated discharge. | □ | □ | □ | □ | □ |
| Prescribe a longer course of antibiotics after discharge if you have concern for readmission shortly after discharge. | □ | □ | □ | □ | □ |

1. Do you believe that you might be overprescribing antibiotics in the inpatient setting?

Check one

- Yes, definitely
- Yes, probably
- Not sure
- Not likely
- No, definitely not

**Antibiotic Prescribing Scenarios**

We would like to know a little more about how providers approach antibiotic prescribing for their inpatients. Please read the following scenarios and answer the questions that follow each one.

**SCENARIO 1:**

A 67 year old man with well controlled Diabetes Mellitus is admitted to your inpatient service complaining of redness and swelling of his left foot, spreading up his leg.  He has no history of chronic foot lesions or ulcers. He is moderately febrile (101.5 degrees Fahrenheit) but vital signs are otherwise stable. He appears non-toxic, with normal mental status.  His left foot is mildly swollen and erythema extends from his foot to the upper calf with lymphangitic streaking.  There is no underlying induration or abscess.  His white cell count is 14,000 (80% neutrophils) and electrolytes are normal.

1. How would you classify the clinical presentation described above?

Check all that apply

- Cellulitis
- Diabetic foot infection
- MRSA infection
- Necrotizing fasciitis
- Other diagnosis (specify): ______________________________________

1. Which antibiotic therapy would you prescribe for this patient?

Check all that apply

- Vancomycin
- Piperacillin-tazobactam
- Cefazolin
- Ceftriaxone
- Clindamycin
- Levofloxacin
- Ertapenem
- Amipicillin-Sulbactam
- Other antibiotic (specify): __________________________________
- No antibiotic

**SCENARIO 1A:**

The patient above was started on appropriate antibiotics and transferred to your care on day 3. His blood cultures were originally positive for group A streptococcus but have since cleared. Your evaluation of the patient indicates that his cellulitis has improved, but he needs another 1-2 days of intravenous antibiotic therapy.

1. Would you change the antibiotic regimen for this patient on day 3?

Check one

- Yes **⇨** *Continue with Q27*
- No **⇨** *Skip to Q28*

1. Which antibiotic regimen would you prescribe at this time?

Check all that apply

- Vancomycin
- Cefazolin
- Penicillin
- Ampicillin
- Piperacillin-tazobactam
- Clindamycin
- Ceftriaxone
- Levofloxacin
- Ertapenem
- Amipicillin-Sulbactam
- Other antibiotic (specify): __________________________________
- No antibiotic

**SCENARIO 1B:**

It is now day 5 since this patient was admitted. He is much improved and is being discharged home.

1. Which antibiotic therapy would you use at this point?

Check all that apply

- Amoxicillin
- Penicillin
- Amoxicillin-clavulanate
- Cephalexin
- Clindamycin
- Dicloxacillin
- Doxycycline
- Levofloxacin
- Linezolid
- Trimethoprim-sulfamethoxazole
- Other antibiotic (specify): __________________________________
- No antibiotics

1. How many *total* days of antibiotic therapy would you utilize for this patient?
   Include inpatient and outpatient days.

Enter #/days

1. If you were to manage this patient, how likely would you be to use the following resources in this patient’s management? If the option does not apply or is not generally available to you, check N/A.

|  | Very likely | Somewhat likely | Not very likely | Not at all likely | N/A |
| --- | --- | --- | --- | --- | --- |
|  | Check one on each line | | | | |
| Pre-specified guidance from facility (e.g., ID specifications, CPRS template) | □ | □ | □ | □ | □ |
| General medical resource, either online or hardcopy (e.g., UpToDate, medical textbook) | □ | □ | □ | □ | □ |
| Sanford guide or other antibiotic prescribing reference, either online or hardcopy | □ | □ | □ | □ | □ |
| Information/Input from an inpatient ward pharmacist | □ | □ | □ | □ | □ |
| Information/Input from another clinician on your inpatient team | □ | □ | □ | □ | □ |
| Information/Input from an ID specialist or ASP team | □ | □ | □ | □ | □ |
| Other resource (specify): ___________ | □ | □ | □ | □ | □ |

1. Without the use of these resources to manage the patient in Scenario 1, how confident would you be in making antibiotic prescribing decisions for this patient?

Check one

- Very confident
- Somewhat confident
- Not very confident
- Not at all confident

**SCENARIO 2**

An 80 year old man is admitted to the general medical ward from home due to increasing shortness of breath and a productive cough and fever.  He has mild dementia, hypertension and prostate hypertrophy. He has no drug allergies.  A chest radiograph indicates left and right lower lobes with patchy opacifications.  He looks moderately ill though not in distress; his lung examination is consistent with the radiographic findings. His temperature is 101°F, blood pressure 110/55, heart rate 104, and respiratory rate 18.  His white cell count is 16 with 85% neutrophils; he has normal renal function and other laboratory tests.  Blood cultures, sputum gram stain and culture are pending.  A rapid influenza test is negative.

1. How would you classify the clinical presentation described above?

Check one

- Health care associated pneumonia
- Community acquired pneumonia
- Aspiration pneumonia
- Sepsis of unclear etiology
- Other etiology (specify___________________________)

1. Which antibiotic therapy would you prescribe for this patient?

Check all that apply

- Vancomycin
- Piperacillin-tazobactam.
- Ceftriaxone
- Azithromycin
- Cefepime
- Levofloxacin
- Linezolid
- Meropenem
- Metronidazole
- Other antibiotic (specify): __________________________________
- No antibiotics

**SCENARIO 2A**

It is now day 3 since the patient was admitted and he is doing well. The patient has negative blood cultures, but a high-quality sputum culture grew Streptococcus pneumoniae that is susceptible to penicillin, azithromycin, and levofloxacin.

1. Which oral antibiotic(s) would you prescribe at this point?

Check all that apply

- Amoxicillin
- Penicillin
- Amoxicillin-clavulanate
- Azithromycin
- Clindamycin
- Doxycycline
- Levofloxacin
- Linezolid
- Trimethoprim-sulfamethoxazole
- Other antibiotic (specify): __________________________________
- No antibiotics

1. How many total days of antibiotic therapy would you utilize for this patient?
   Include inpatient and outpatient days.
2. For a patient similar to the one described above, how likely would you be to use the following resources, in this patient’s management? If the option does not apply or is not generally available to you, check N/A.

|  | Very likely | Somewhat likely | Not very likely | Not at all likely | N/A |
| --- | --- | --- | --- | --- | --- |
|  | Check one on each line | | | | |
| Pre-specified guidance from facility (e.g., ID specifications, CPRS template) | □ | □ | □ | □ | □ |
| General medical resource, either online or hardcopy (e.g., UpToDate, medical textbook) | □ | □ | □ | □ | □ |
| Sanford guide or other antibiotic prescribing reference, either online or hardcopy | □ | □ | □ | □ | □ |
| Information/Input from an inpatient ward pharmacist | □ | □ | □ | □ | □ |
| Information/Input from another clinician on your inpatient team | □ | □ | □ | □ | □ |
| Information/Input from an ID specialist or ASP team | □ | □ | □ | □ | □ |
| Other resource (specify): ___________ | □ | □ | □ | □ | □ |

1. Without the use of these resources to manage the patient in Scenario 2, how confident would you be in making antibiotic prescribing decisions for this patient?

Check one

- Very confident
- Somewhat confident
- Not very confident
- Not at all confident

**SCENARIO 3:**

A 72 year-old male with diabetes mellitus controlled on oral medications has a urinalysis sent upon admission for chest pain. He denies frequency, urgency, flank pain, or fever but notes chronic nocturia (~2x/night, unchanged over the past several years). The urinalysis shows 17 WBCs/HPF, 5 RBCs/HPF, and positive leukocyte esterase and nitrite. A urine culture is reflexively sent that grows >100,000 colonies of E. coli that is susceptible to all β-lactams, cephalosporins, fluoroquinolones, trimethoprim-sulfamethoxazole, and nitrofurantoin.

1. How would you classify the clinical presentation described above?

Check all that apply

- Uncomplicated urinary tract infection
- Complicated urinary tract infection
- Asymptomatic bacteriuria
- Other diagnosis (specify): ______________________________________

1. Which antibiotic therapy would you prescribe for this patient?

Check all that apply

- Ciprofloxacin 500mg orally twice daily for 7 days
- Trimethoprim-sulfamethoxazole 1 DS tablet orally twice daily for 10 days
- Cephalexin 500mg orally twice daily for 14 days
- Other antibiotic (specify): __________________________________
- No antibiotics

1. If you were to manage this patient, how likely would you be to use the following resources in this patient’s management? If the option does not apply or is not generally available to you, check N/A.

|  | Very likely | Somewhat likely | Not very likely | Not at all likely | N/A |
| --- | --- | --- | --- | --- | --- |
|  | Check one on each line | | | | |
| Pre-specified guidance from facility (e.g., ID specifications, CPRS template) | □ | □ | □ | □ | □ |
| General medical resource, either online or hardcopy (e.g., UpToDate, medical textbook) | □ | □ | □ | □ | □ |
| Sanford guide or other antibiotic prescribing reference, either online or hardcopy | □ | □ | □ | □ | □ |
| Information/Input from an inpatient ward pharmacist | □ | □ | □ | □ | □ |
| Information/Input from another clinician on your inpatient team | □ | □ | □ | □ | □ |
| Information/Input from an ID specialist or ASP team | □ | □ | □ | □ | □ |
| Other resource (specify): ___________ | □ | □ | □ | □ | □ |

1. Without the use of these resources to manage the patient in Scenario 3, how confident would you be in making antibiotic prescribing decisions for this patient?

Check one

- Very confident
- Somewhat confident
- Not very confident
- Not at all confident

**SCENARIO 4:**

An 87 year-old male nursing home resident with mild dementia and an indwelling Foley catheter is seen for increasing agitation of one day’s duration.  He was recently prescribed diphenhydramine for pruritis.  He is afebrile with normal vital signs.  He denies flank or abdominal pain, and on physical exam, there is no abdominal or suprapubic tenderness.  Urinalysis shows 56 WBCs/HPF, 17 RBCs/HPF, and positive leukocyte esterase and nitrite.  A urine culture grows >100,000 colonies of E. coli that is resistant to fluoroquinolones and trimethoprim-sulfamethoxazole but susceptible to cephalexin, amoxicillin-clavulanate, and nitrofurantoin.  Estimated creatinine clearance is 70 mL/min.

1. How would you classify the clinical presentation described above?

Check all that apply

- Uncomplicated catheter-associated urinary tract infection
- Complicated catheter-associated urinary tract infection
- Asymptomatic bacteriuria
- Other diagnosis (specify): ______________________________________

1. Which antibiotic therapy would you prescribe for this patient? (Assume catheter has been changed)

Check all that apply

- Ceftriaxone 1gm IV once daily with transition to cephalexin when mental status returns to baseline, complete at least 7 days
- Amoxicillin-clavulanate 500/125mg orally twice daily for 14 days
- Nitrofurantoin 100mg orally four times daily for 7 days
- Other antibiotic and/or antibiotic regimen_______(list)
- No antibiotics

1. If you were to manage this patient, how likely would you be to use the following resources, in this patient’s management? If the option is not applicable or not generally available to you, check N/A.

|  | Very likely | Somewhat likely | Not very likely | Not at all likely | N/A |
| --- | --- | --- | --- | --- | --- |
|  | Check one on each line | | | | |
| Pre-specified guidance from facility (e.g., ID specifications, CPRS template) | □ | □ | □ | □ | □ |
| General medical resource, either online or hardcopy (e.g., UpToDate, medical textbook) | □ | □ | □ | □ | □ |
| Sanford guide or other antibiotic prescribing reference, either online or hardcopy | □ | □ | □ | □ | □ |
| Information/Input from an inpatient ward pharmacist | □ | □ | □ | □ | □ |
| Information/Input from another clinician on your inpatient team | □ | □ | □ | □ | □ |
| Information/Input from an ID specialist or ASP team | □ | □ | □ | □ | □ |
| Other resource (specify): ___________ | □ | □ | □ | □ | □ |

1. Considering all the resources available to you to help mange the patient in Scenario 4, how confident would you be in making antibiotic prescribing decisions for this patient?

Check one

- Very confident
- Somewhat confident
- Not very confident
- Not at all confident

**Antibiotic Prescribing Resources**

These next questions are about resources potentially available to you in the past 12 months regarding antibiotic prescribing for patients who are admitted with a diagnosis of either pneumonia (PNA), skin and soft tissue infection (SSTI), or urinary tract infections (UTI).

1. In the past 12 months, did your facility’s ID or ASP team provide any new *general* guidance for the following decision points in conjunction with the following conditions?

|  | IF yes, check the corresponding box on each line | |
| --- | --- | --- |
| The initial *choice* of antibiotics used to treat: | | |
| - PNA | - SSTI | - UTI |
| *Changing* *or tailoring* the antibiotic regimen after ±3 days to treat: | | |
| - PNA | - SSTI | - UTI |
| The *completion* *or duration* of the antibiotic treatment course for: | | |
| - PNA | - SSTI | - UTI |

1. Did this guidance impact your antibiotic prescribing practices?
   If there was no guidance on a particular practice, leave blank.

|  | Yes | No |
| --- | --- | --- |
|  | Check one on each line | |
| The initial *choice* of antibiotics used to treat: | | |
| **PNA** | □ | □ |
| **SSTI** | □ | □ |
| **UTI** | □ | □ |
| *Changing* *or tailoring* the antibiotic regimen after ±3 days to treat: | | |
| **PNA** | □ | □ |
| **SSTI** | □ | □ |
| **UTI** | □ | □ |
| The *completion* *or duration* of the antibiotic treatment course for: | | |
| **PNA** | □ | □ |
| **SSTI** | □ | □ |
| **UTI** | □ | □ |

1. Over the past 12 months, did you regularly use any of the following tools in making antibiotic prescribing decisions?

Check all that apply

- Antibiogram
- CPRS templates or menus
- ID intra or internet site
- UpToDate or other online general reference
- Sanford guide or other antibiotic reference
- Other tool (specify): __________________
- None of the above

1. Over the past 12 months, were any of the following mechanisms available at your VA to help you improve your own antibiotic prescribing practices?

Check all that apply

- Antibiotic prescribing dashboard showing general or patient level stats
- Antibiotic use measures or other metrics (antimicrobial resistance patterns)
- Audit and feedback
- Other feedback mechanisms (specify): _______________________
- No mechanisms available

1. How helpful were these mechanisms in improving your antibiotic prescribing practices for your inpatients? If you did not use a particular mechanism, check N/A.

|  | Extremely helpful | Very helpful | Moderately helpful | Minimally helpful | Not helpful | N/A |
| --- | --- | --- | --- | --- | --- | --- |
|  | Check one on each line | | | | |  |
| Antibiotic prescribing electronic dashboard | □ | □ | □ | □ | □ | □ |
| Antibiotic use measures or other metrics | □ | □ | □ | □ | □ | □ |
| Audit and feedback | □ | □ | □ | □ | □ | □ |
| Other feedback mechanisms (specify): ______________________________ | □ | □ | □ | □ | □ | □ |

1. At your facility, how much of an obstacle or benefit are antibiotic stewardship programs to good patient care?

Check one

- Considerable obstacle
- Moderate obstacle
- Minor obstacle
- Neither obstacle nor benefit
- Minor benefit
- Moderate benefit
- Considerable benefit
- Don’t know

1. Which of the following people have you interacted with from your facility’s antimicrobial stewardship program (ASP)?

Check all that apply

- ID Attending(s)
- ID Pharmacist(s)
- ID Fellow(s)
- Pharmacy residents
- Non-ID Physicians
- Other role(s): ___________________________
- Not familiar with facility’s ASP

1. What is your overall satisfaction with the assistance you have received from your VA regarding antibiotic prescribing for your inpatients over the past 12 months? Include any kind of assistance (e.g., consults, tools, education, feedback, informational resources).

Check one

- Very satisfied
- Satisfied
- Neither satisfied nor dissatisfied
- Dissatisfied
- Very dissatisfied

1. How helpful would the following be improving your antibiotic prescribing?

|  | Extremely helpful | Very helpful | Moderately helpful | Not very helpful | Not at all helpful |
| --- | --- | --- | --- | --- | --- |
|  | Check one on each line | | | | |
| Additional feedback on your antibiotic selections | □ | □ | □ | □ | □ |
| Additional education or guidance on antibiotic use | □ | □ | □ | □ | □ |
| Additional CPRS or other electronic templates to guide antibiotic prescribing | □ | □ | □ | □ | □ |
| Additional resources (specify): | □ | □ | □ | □ | □ |

**Personal Characteristics**

1. What is your age?

Check one

25 or under

26-35

36-45

Over 45

1. What is your gender?

Check one

Male

Female

**Thank you for participating in this study!**

| **Comments (optional):** |
| --- |
