# Supplementary material for: Performance of infectious diseases specialists, hospitalists, and other internal medicine physicians in antimicrobial case-based scenarios: Potential impact of antimicrobial stewardship programs at 16 Veterans’ Affairs medical centers
Source: Infect Control Hosp Epidemiol. 2022 May 4;44(3):400–5. doi: 10.1017/ice.2022.100 (PMC10015262; doi:10.1017/ice.2022.100)
Supplement: Supplementary file 1 [file S0899823X22001003sup.zip › S0899823X22001003sup002.docx]

**Supplement B: Supplemental Tables**

**Supplemental Table 1: Antibiotic prescribing practices and resource utilization among survey respondents (by specialty)**

|  | **ID (n=19)** | **Hospitalists (n=62)** | **Other Internists (n=58)** |
| --- | --- | --- | --- |
| *Antibiotic prescribing practices:* | | | |
| *How confident are you that you use antibiotics optimally in the inpatient setting?* | | | |
| Very confident, n (%)** | 13 (68.4%) | 10 (16.1%) | 6 (10.3%) |
| Somewhat confident, n (%) | 5 (26.3%) | 49 (79%) | 46 (79.3%) |
| Not very/not at all confident, n (%) | 1 (5.3%) | 3 (4.8%) | 5 (8.6%) |
| *In general, for hospitalized patients who are admitted with a diagnosis of either uncomplicated healthcare-acquired pneumonia, non-diabetic cellulitis, or uncomplicated UTI (cystitis), how important do you feel it is to reassess the antibiotic regimen on or around day 3 to tailor therapy?* | | | |
| Very important, n (%) | 18 (94.7%) | 50 (80.6%) | 54 (93.1%) |
| Somewhat important, n (%) | 1 (5.3%) | 11 (17.7%) | 4 (6.9%) |
| Not very/not at all important, n (%) | 0 (0%) | 1 (1.6%) | 0 (0%) |
| *For patients that were admitted to your care for an infectious etiology during the past 12 months, how often did you do the following?* (those answering “Always/most often (81-100%)” or “Usually” (61-80%) are tabulated) | | | |
| Change the antibiotics that were initially started by the ER (usually on day 1 or 2 of hospitalization). That is, adjust the antibiotic regimen soon after admission. n (%) | 14 (73.7%) | 36 (58.1%) | 31 (53.4%) |
| Tailor the antibiotic regimen on or around day 3, if appropriate. That is, narrow the antibiotic regimen after the patient has been hospitalized for several days. n (%) | 16 (84.2%) | 48 (77.4%) | 40 (69%) |
| Observe the patient on oral antibiotics for at least one day before discharge. n (%) | 2 (10.5%) | 13 (21%) | 11 (19%) |
| Continue with an IV antibiotic until within a day of anticipated discharge. n (%) | 4 (21.1%) | 25 (40.3%) | 17 (29.3%) |
| Prescribe a longer course of antibiotics after discharge if you have concern for readmission shortly after discharge. n (%) | 1 (5.3%) | 6 (9.7%) | 2 (3.4%) |
| *Do you believe that you might be overprescribing antibiotics in the inpatient setting?* | | | |
| Yes, definitely, n (%) | 1 (5.3%) | 2 (3.2%) | 0 (0%) |
| Yes, probably, n (%) | 4 (21.1%) | 33 (53.2%) | 22 (37.9%) |
| Not sure, n (%) | 4 (21.1%) | 12 (19.4%) | 21 (36.2%) |
| No, not likely, n (%)* | 8 (42.1%) | 12 (19.4%) | 15 (25.9%) |
| No, definitely not, n (%) | 2 (10.5%) | 3 (4.8%) | 0 (0%) |
| *Over the past 12 months, did you regularly use any of the following tools in making antibiotic prescribing decisions?* | | | |
| Antibiogram, n (%)* | 16 (84.2%) | 45 (72.6%) | 31 (53.4%) |
| Electronic health record templates or menus, n (%) | 3 (15.8%) | 25 (40.3%) | 21 (36.2%) |
| Local ID intranet/internet site, n (%) | 8 (42.1%) | 16 (25.8%) | 12 (20.7%) |
| UpToDate or other online general reference, n (%) | 15 (78.9%) | 52 (83.9%) | 45 (77.6%) |
| Sanford guide or other antibiotic reference, n (%) | 11 (57.9%) | 27 (43.5%) | 19 (32.8%) |
| *How helpful would the following be in improving your antibiotic prescribing?* | | | |
| Additional feedback on your antibiotic selections, “extremely/very helpful” (%)* | 10 (52.6%) | 51 (82.3%) | 42 (72.4%) |
| Additional education or guidance on antibiotic prescribing, “extremely/very helpful” (%)** | 5 (26.3%) | 46 (74.2%) | 27 (46.6%) |
| Additional electronic health record or other electronic templates to guide antibiotic prescribing, “extremely/very helpful” (%) | 6 (31.6%) | 33 (53.2%) | 33 (56.9%) |

*: p <0.05

**: p <0.001

**Supplemental Table 2: Utilization of ID/antimicrobial stewardship team guidance in prior 12 months, by clinical condition (n=120 non-ID physicians)**

|  | **Pneumonia** | **Skin-Soft Tissue Infection** | **Urinary Tract Infection** |
| --- | --- | --- | --- |
| *Did your facility’s ID or antimicrobial stewardship team provide any new general guidance for the following decision points in conjunction with the following conditions?* | | | |
| Initial choice of antibiotics, n (%) | 40 (33.3%) | 32 (26.7%) | 34 (28.3%) |
| Changing or tailoring the antibiotic regimen after approximately 3 days, n (%) | 35 (29.2%) | 30 (25%) | 30 (25%) |
| The completion or duration of the antibiotic treatment course, n (%) | 35 (29.2%) | 32 (26.7%) | 35 (29.2%) |
| *Did this guidance impact your antibiotic prescribing practices?* | | | |
| Yes, at initial choice of antibiotics, n (%) | 35 (87.5%) | 25 (78.1%) | 26 (76.5%) |
| Yes, at changing or tailoring after approximately 3 days, n (%)* | 34 (97.1%) | 24 (80%) | 27 (90%) |
| Yes, at completion or duration of antibiotic treatment course, n (%) | 32 (91.4%) | 30 (93.8%) | 30 (85.7%) |

*: p <0.05

**Supplemental Table 3: Clinical scenario responses by practice type**†

|  | **ID (n=19)** | **Hospitalists (n=62)** | **Other Internists (n=58)** |
| --- | --- | --- | --- |
| *Scenario 1: A 67 year-old man with well controlled diabetes mellitus is admitted to your inpatient service complaining of redness and swelling of his left foot, spreading up his leg. He has no history of chronic foot lesions or ulcers. He is moderately febrile (101.5˚ Fahrenheit) but vital signs are otherwise stable. He appears non-toxic, with normal mental status. His left foot is mildly swollen and erythema extends from his foot to the upper calf with lymphangitic streaking. There is no underlying induration or abscess. His white cell count is 14,000 (80% neutrophils) and electrolytes are normal.* | | | |
| *How would you classify the clinical presentation described above?* | | | |
| Most guideline-concordant (cellulitis) (+1 point), n (%)* | 19 (100%) | 43 (69.4%) | 40 (69%) |
| Less guideline-concordant (diabetic foot infection) (0 points), n (%) | 0 (0%) | 15 (24.2%) | 14 (24.1%) |
| Guideline-discordant (MRSA infection, necrotizing fasciitis) (-1 point), n (%) | 0 (0%) | 3 (4.8%) | 0 (0%) |
| *Which antibiotic therapy would you prescribe for this patient?* | | | |
| Most guideline-concordant (cefazolin) (+1 point), n (%) | 10 (52.6%) | 24 (38.7%) | 23 (39.7%) |
| Less guideline-concordant (vancomycin, ceftriaxone, clindamycin, ampicillin-sulbactam, cephalexin) (0 points), n (%) | 9 (47.4%) | 26 (41.9%) | 17 (29.3%) |
| Guideline-discordant (piperacillin-tazobactam, levofloxacin, ertapenem, no antibiotic) (-1 point), n (%) | 0 (0%) | 11 (17.7%) | 13 (22.4%) |
| *The patient above was started on appropriate antibiotics and transferred to your care on day 3. His blood cultures were originally positive for group A streptococcus but have since cleared. Your evaluation of the patient indicates that his cellulitis has improved, but he needs another 1-2 days of intravenous antibiotic therapy. Which antibiotic regimen would you prescribe at this time?* | | | |
| Most guideline-concordant (cefazolin, penicillin, ampicillin, cephalexin) (+1 point), n (%) | 16 (84.2%) | 42 (67.7%) | 27 (46.5%) |
| Less guideline-concordant (ceftriaxone, clindamycin, ampicillin-sulbactam) (0 points), n (%) | 3 (15.8%) | 17 (27.4%) | 24 (41.4%) |
| Guideline-discordant (vancomycin, piperacillin-tazobactam, levofloxacin, ertapenem, no antibiotic) (-1 point), n (%) | 0 (0%) | 2 (3.2%) | 1 (1.7%) |
| *It is now day 5 since this patient was admitted. He is much improved and is being discharged home. Which antibiotic therapy would you use at this point?* | | | |
| Most guideline-concordant (amoxicillin, cephalexin, penicillin, dicloxacillin, no antibiotic) (+1 point), n (%) | 16 (84.2%) | 46 (74.2%) | 33 (56.9%) |
| Less guideline-concordant (amoxicillin-clavulanate, clindamycin) (0 points), n (%) | 3 (15.8%) | 12 (19.4%) | 17 (29.3%) |
| Guideline-discordant (doxycycline, levofloxacin, linezolid, trimethoprim-sulfamethoxazole) (-1 point), n (%) | 0 (0%) | 2 (3.2%) | 3 (5.2%) |
| *How many total days of antibiotic therapy would you utilize for this patient?* | | | |
| Most guideline-concordant (5-10 days) (+1 point), n (%) | 11 (57.9%) | 42 (67.7%) | 36 (62.1%) |
| Less guideline-concordant (11-14 days) (0 points), n (%) | 7 (36.8%) | 18 (29%) | 13 (22.4%) |
| Guideline-discordant (0-4 days) (-1 point), n (%) | 0 (0%) | 0 (0%) | 1 (1.7%) |
|  | | | |
| *Scenario 2: An 80 year-old man is admitted to the general medical ward from home due to increasing shortness of breath and a productive cough and fever. He has mild dementia, hypertension and prostate hypertrophy. He has no drug allergies. A chest radiograph indicates left and right lower lobes with patchy opacifications. He looks moderately ill though not in distress; his lung examination is consistent with the radiographic findings. His temperature is 101°F, blood pressure 110/55, heart rate 104, and respiratory rate 18. His white cell count is 16 with 85% neutrophils; he has normal renal function and other laboratory tests. Blood cultures, sputum gram stain and culture are pending. A rapid influenza test is negative.* | | | |
| *How would you classify the clinical presentation described above?* | | | |
| Most guideline-concordant (community-acquired pneumonia) (+1 point), n (%) | 15 (79%) | 43 (69.4%) | 41 (70.7%) |
| Less guideline-concordant (aspiration pneumonia) (0 points), n (%) | 2 (10.5%) | 10 (16.1%) | 8 (13.8%) |
| Guideline-discordant (healthcare-associated pneumonia, sepsis of unclear etiology) (-1 point), n (%) | 1 (5.3%) | 5 (8.1%) | 1 (1.7%) |
| *Which antibiotic therapy would you prescribe for this patient?* | | | |
| Most guideline-concordant (ceftriaxone, azithromycin, ampicillin-sulbactam, doxycycline) (+1 point), n (%) | 14 (73.7%) | 41 (66.1%) | 34 (58.6%) |
| Less guideline-concordant (levofloxacin, metronidazole) (0 points), n (%) | 1 (5.3%) | 15 (17.7%) | 12 (20.7%) |
| Guideline-discordant (vancomycin, piperacillin-tazobactam, cefepime, linezolid, meropenem, no antibiotic) (-1 point), n (%) | 3 (15.8%) | 6 (9.7%) | 4 (6.9%) |
| *It is now day 3 since the patient was admitted and he is doing well. The patient has negative blood cultures, but a high-quality sputum culture grew Streptococcus pneumoniae that is susceptible to penicillin, azithromycin, and levofloxacin. Which oral antibiotic(s) would you prescribe at this point?* | | | |
| Most guideline-concordant (amoxicillin, penicillin, cephalexin) (+1 point), n (%)* | 14 (73.7%) | 21 (33.9%) | 19 (32.8%) |
| Less guideline-concordant (amoxicillin-clavulanate, azithromycin, clindamycin, doxycycline, levofloxacin, cefdinir) (0 points), n (%) | 4 (21.1%) | 37 (59.7%) | 31 (53.4%) |
| Guideline-discordant (linezolid, trimethoprim-sulfamethoxazole, no antibiotic) (-1 point), n (%) | 0 (0%) | 0 (0%) | 0 (0%) |
| *How many total days of antibiotic therapy would you utilize for this patient?* | | | |
| Most guideline-concordant (5-7 days) (+1 point), n (%) | 17 (89.5%) | 55 (88.7%) | 40 (69%) |
| Less guideline-concordant (8-14 days) (0 points), n (%) | 0 (0%) | 3 (4.8%) | 8 (13.8%) |
| Guideline-discordant (0-4 days) (-1 point), n (%) | 0 (0%) | 0 (0%) | 0 (0%) |
|  |  |  |  |
| *Scenario 3: A 72 year old male with diabetes mellitus controlled on oral medications has a urinalysis sent upon admission for chest pain. He denies frequency, urgency, flank pain, or fever but notes chronic nocturia (~2x/night, unchanged over the past several years). The urinalysis shows 17 WBCs/HPF, 5 RBCs/HPF, and positive leukocyte esterase and nitrite. A urine culture is reflexively sent that grows >100,000 colonies of E. coli that is susceptible to all β-lactams, cephalosporins, fluoroquinolones, trimethoprim-sulfamethoxazole, and nitrofurantoin.* | | | |
| *How would you describe the clinical presentation described above?* | | | |
| Most guideline-concordant (asymptomatic bacteriuria) (+1 point), n (%) | 15 (79%) | 47 (75.8%) | 38 (65.5%) |
| Guideline-discordant (uncomplicated urinary tract infection, complicated urinary tract infection) (-1 point), n (%) | 3 (15.8%) | 12 (19.4%) | 14 (24.1%) |
| *Which antibiotic would you prescribe for this patient?* | | | |
| Most guideline-concordant (no antibiotic) (+1 point), n (%)* | 15 (79%) | 46 (74.2%) | 35 (60.3%) |
| Guideline-discordant (ciprofloxacin, trimethoprim-sulfamethoxazole, cephalexin, amoxicillin) (-1 point), n (%) | 3 (15.8%) | 14 (22.5%) | 15 (25.8%) |
|  | | | |
| *Scenario 4: An 87 year old male nursing home resident with mild dementia and an indwelling Foley catheter is seen for increasing agitation of one day’s duration. He was recently prescribed diphenhydramine for pruritis. He is afebrile with normal vital signs. He denies flank or abdominal pain, and on physical exam, there is no abdominal or suprapubic tenderness. Urinalysis shows 56 WBCs/HPF, 17 RBCs/HPF, and positive leukocyte esterase and nitrite. A urine culture grows >100,000 colonies of E. coli that is resistant to fluoroquinolones and trimethoprim-sulfamethoxazole but susceptible to cephalexin, amoxicillin-clavulanate, and nitrofurantoin. Estimated creatinine clearance is 70 mL/min.* | | | |
| *How would you describe the clinical presentation described above?* | | | |
| Most guideline-concordant (asymptomatic bacteriuria) (+1 point), n (%) | 9 (47.4%) | 23 (37.1%) | 19 (32.8%) |
| Guideline-discordant (uncomplicated catheter-associated urinary tract infection, complicated catheter-associated urinary tract infection) (-1 point), n (%) | 9 (47.4%) | 34 (54.8%) | 31 (53.5%) |
| *Which antibiotic would you prescribe for this patient?* | | | |
| Most guideline-concordant (no antibiotic) (+1 point), n (%) | 14 (73.7%) | 29 (46.8%) | 24 (41.4%) |
| Guideline-discordant (ceftriaxone, amoxicillin-clavulanate, nitrofurantoin, cephalexin) (-1 point), n (%) | 4 (21.1%) | 28 (45.2%) | 27 (46.6%) |

*: p <0.05

†: As not all respondents completed all questions, percentages do not necessarily add up to 100% for each physician category and question

**Supplemental Table 4: Resources Likely to Be Used in Patient Management Across Clinical Scenarios**

|  | Scenario 1 | Scenario 2 | Scenario 3 | Scenario 4 |
| --- | --- | --- | --- | --- |
| Pre-specified guidance from facility (e.g., ID specification, electronic health record template), n (%) | 51 (36.4%) | 49 (35%) | 35 (25%) | 37 (26.4%) |
| General medical resource, either online or hardcopy (e.g., UpToDate, medical textbook), n (%) | 64 (45.7%) | 52 (37.1%) | 47 (33.6%) | 48 (34.3%) |
| Sanford guide or other antibiotic prescribing reference, either online or hardcopy, n (%) | 37 (26.4%) | 31 (22.1%) | 23 (16.4%) | 23 (16.4%) |
| Information/input from an inpatient ward pharmacist, n (%) | 58 (41.4%) | 41 (29.3%) | 35 (25%) | 29 (20.7%) |
| Information/input from another clinician on your inpatient team, n (%) | 28 (20%) | 26 (18.6%) | 22 (15.7%) | 24 (17.1%) |
| Information/input from an ID specialist or antimicrobial stewardship team | 50 (35.7%) | 37 (26.4%) | 31 (22.1%) | 34 (24.3%) |

**Supplemental Table 5: Confidence in antibiotic prescribing decisions, by scenario and specialty**

| **Respondents answering “Very confident”** | **ID (n=19)** | **Hospitalists (n=62)** | **Other Internists (n=58)** |
| --- | --- | --- | --- |
| Scenario 1 (cellulitis), n (%)** | 16 (84.2%) | 17 (27.4%) | 23 (39.7%) |
| Scenario 2 (community-acquired pneumonia), n (%)* | 14 (73.7%) | 26 (41.9%) | 26 (44.8%) |
| Scenario 3 (non-catheter-associated asymptomatic bacteriuria), n (%)* | 14 (73.7%) | 41 (66.1%) | 25 (43.1%) |
| Scenario 4 (catheter-associated asymptomatic bacteriuria), n (%)* | 12 (63.2%) | 20 (32.3%) | 17 (29.3%) |

*: p <0.05

**: p <0.001
